# Traditional statistics and artificial intelligence-based prognostic models for predicting type 2 diabetes mellitus after gestational diabetes: a systematic review

**DOI:** 10.1186/s41512-026-00229-8

**Published:** 2026-04-20

**Authors:** Nigus Bililign Yimer, Yitayeh Belsti, Beminate Lemma Seifu, Rebecca Goldstein, Helena Teede, Joanne Enticott

**Affiliations:** 1 Monash Centre for Health Research and Implementation, Faculty of Medicine, Nursing and Health Sciences, Monash University, 43-51 Kanooka Grove 3138, Melbourne, VIC Australia; 2 Diabetes and Endocrine Units, Monash Health, Melbourne, Australia

**Keywords:** Artificial intelligence, Gestational diabetes mellitus, Machine learning, Risk prediction, Systematic review, Prognostic model, Type 2 diabetes mellitus

## Abstract

**Background:**

Women with gestational diabetes (GDM) are at increased risk of developing type 2 diabetes (T2D). Prognostic models have been developed and evaluated, but their methodological quality and applicability remain inconclusive. Recent reviews with the latest search date of March 31, 2025, were conducted with major shortcomings, including failure to adhering best-practice guides and inappropriately pooling heterogeneous model performances. This systematic review aims to synthesise the methodological characteristics of existing prognostic models for T2D following GDM.

**Methods:**

Five electronic databases were searched from inception to January 24, 2026. Prognostic models predicting T2D following GDM, regardless of study setting were included. Data extraction adhered to existing expert guidelines. Quality and applicability were assessed using the updated Prediction model Risk Of Bias ASsessment tool. Two reviewers independently screened and assessed quality, resolving disagreements through consensus and involvement of a third reviewer.

**Results:**

Our updated review identified six more studies than the previous review. Our review identified 19 studies with 20 models, half from prospective cohorts (*n* = 18) mostly in hospital settings across North America, Europe, Australia, and Asia. Logistic regression model was most common (*n* = 9), followed by machine learning (*n* = 6), and Cox regression (*n* = 5). Internal and external validation were done in only 13 and 1 models, respectively. Discrimination was widely reported (Area Under the Curve (AUC) 0.67–0.92); while calibration, overall performance, and clinical utility measures were underreported. Only one study reported appropriate sample size determination. Maternal age, pregnancy fasting glucose, and BMI were common predictors. Risk of bias was generally low during development and evaluation phases, but applicability concerns were high in 60% of models.

**Conclusions:**

While several models demonstrated acceptable performance and low concern in selected quality domains, generalizability and clinical utility remain limited due to high concerns in applicability and inconsistent reporting. Adherence to best practice guides such as TRIPOD + AI, external validation of models, and exploration of novel prediction modelling techniques are recommended to advance the reporting and application of risk tools for personalised medicine post GDM. This review is the first to apply the PROBAST + AI, enabling a comprehensive evaluation of quality and applicability compared to previous works in the field.

**Protocol registration:**

PROSPERO CRD420251034657.

**Supplementary Information:**

The online version contains supplementary material available at 10.1186/s41512-026-00229-8.

## Background

In 2021, the global standardised prevalence of gestational diabetes mellitus (GDM) was reported to be 14.0% [1]. Regional estimates varied significantly, with 27.6% in Middle East and North Africa, 20.8% in Southeast Asia, and 14.7% in Western Pacific [1]. GDM is defined as hyperglycaemia that develops during pregnancy in a previously normoglycemic woman [2]. GDM carries a high risk of adverse outcomes in pregnancy and substantially higher risk of developing type 2 diabetes (T2D) after pregnancy [3, 4]. Evidence shows a nearly 10-fold higher risk of developing T2D in this population, compared to women without a prior diagnosis of GDM [5]. T2D poses a significant public health burden with increasing prevalence, morbidities and mortality [6]. The risk of developing postnatal T2D varies depending on individual clinical and demographic characteristics, many of which are modifiable. This enables prevention with the early postpartum period being a critical time to tailor interventions to limit T2D onset [5]. Early identification through risk prediction identifies those who require both targeted postpartum T2D screening and prevention measures [7].

T2D risk prediction models following GDM combine multiple prognostic factors to predict individualised risk and support shared decision making [8]. When appropriately developed and validated, these models have the potential to inform patients and care providers in clinical care and can lead to improved patient care and outcomes [9–11]. Traditionally, logistic regression and other conventional statistical approaches have been widely used to develop such models [12–15]. However, recent advances include machine learning (ML) techniques, and deep learning, offering improved predictive performance and flexibility in handling high-dimensional data [16, 17]. More recently, hybrid models have been developed, integrating ML algorithms that learn patterns from data to make predictions using causal inference approaches. This aims to estimate the effect of exposures or interventions on outcomes and have shown promising effect in advancing ML methods [18, 19].

There are existing models for postnatal T2D risk prediction that use simple and easily accessible variables such as maternal age, BMI, fasting glucose levels, and family history of diabetes [20–23]. However, these models have serious methodological shortcomings. A systematic review revealed poor reporting of important components including predictor selection, model calibration and clinical utility in such models [8]. In prediction models generally, common challenges include inconsistencies in model development and evaluation, small sample sizes and risk of overfitting, limited external validation, underreporting of key performance measures, and lack of adherence to best-practice methods [8, 24–27]. Even in externally validated studies, some argue that prediction models may not truly be validated due to heterogeneity in model performance across geographic locations and settings, and change in populations and predictor measurements over time [28]. Hence, few including postpartum T2D models are implemented into clinical settings or have evidence of clinical impact [27].

While experts in the field empathise using best-practice methods for reporting [10, 29], a recently published systematic review and meta-analysis on the predictive value of ML models for T2D post GDM [30] did not adhere to established best-practice methods, including the Transparent Reporting of a multivariable prediction model of Individual Prognosis Or Diagnosis checklist for Systematic Reviews and Meta-Analysis (TRIPOD-SRMA) and CHecklist for critical Appraisal and data extraction for systematic reviews of prediction Modelling Studies (CHARMS) [31, 32]. Critically, meta-analysis was inappropriately applied to combine predictive performances from widely heterogenous models in terms of algorithms used and predictors included, without accounting for the lack of sufficient externally validated models and between-study variation assumptions. Furthermore, the included models in the meta-analysis widely varied by number and mix of predictors and follow-up durations, and two ineligible studies were included - one that examined a general pregnant population and, another evaluated postpartum glucose screening attendance as the outcome [30]. Another review also repeated similar concerns [33]. These limitations undermined the relevance of the findings, as we have previously highlighted (*unpublished results*). Furthermore, the last literature search was until March 31, 2025, making it timely to seek new studies.

In this context and given the growing number of traditional and artificial intelligence-based risk prediction models, a comprehensive synthesis of methodological quality, performance, applicability, and reporting is warranted to guide screening and personalised prevention for those at risk of T2D. Importantly, no prior review has applied PROBAST + AI or the TRIPOD-SRMA guideline, nor compared statistical and AI-based tools using a comprehensive appraisal system. This review explicitly addresses these significant gaps. This review aims to systematically identify T2D prognostic models developed and/or evaluated for women with previous GDM, appraise their methodologies, summarise performance measures, and investigate reporting standards against available best-practice guidelines.

## Methods

The protocol for this systematic review was registered in the International Prospective Register of Systematic Reviews (PROSPERO CRD420251034657). This work was designed in accordance with the CHARMS tool and existing expert guidance [29, 31]. The TRIPOD-SRMA was adhered throughout the review process [32]. The completed checklist can be found here (Table S1). Noting major methodological limitations in recent reviews [30, 33], we conducted this systematic review to strengthen the evidence.

### Search strategy

The electronic databases searched include MEDLINE(R), EMBASE, Emcare, CINAHL, and SCOPUS from inception to May 12, 2025. After completing screening and preliminary synthesis of initial search results, we conducted updated search up to January 24, 2026 to identify any newly published eligible studies. Building on existing search strategies for prognostic models [34–37], we used the relevant Medical Subject Heading (MeSH) terms and keywords for searching. Additionally, reference lists of selected articles were manually searched. The search was restricted English language articles. An experienced Monash University librarian was consulted about the search strategy. The detailed search strategy can be found here (Table S2).

### Inclusion and exclusion criteria

Any statistical and AI/ML-based studies predicting the risk of T2D in women previously diagnosed with GDM (prognostic models), with any duration of follow-up were included, regardless of the setting and timing. Diagnostic models, commentaries, editorials, letters, narratives, case reports, or expert opinions were excluded. The PICOTS framework was followed (Table 1).

**Table 1:** The PICOTS framework for prognostic models predicting T2D in women with history of GDM

P	Population	Women previously diagnosed with GDM during pregnancy.
I	Index model(s)	All developed prognostic models for T2D after GDM and their corresponding external validation.
C	Comparator model(s)	No predefined comparator.
O	Outcome(s)	Risk of T2D after GDM
T	Timing	The starting point of prediction can be during pregnancy or after delivery, typically at 6 to 12 weeks of postpartum. The follow-up period includes any duration until the diagnosis of T2D.
S	Setting	Not specified; any setting

### Data extraction strategy

For eligibility, articles were screened based on titles and abstracts, followed by full-text screening of eligible articles. The screening process was conducted in Covidence Systematic Review Tool (www.covidence.org). Two reviewers (NBY and BLS) independently screened the articles, and any disagreement was resolved through consensus and involving a third reviewer (YB). Data extraction sheet was prepared in an excel spreadsheet in accordance with the CHARMS tool. Using the defined PICOTS framework, the sheet included information of included studies, including study ID (author and year), source of data, participants (e.g., recruitment, eligibility, setting), region or country, model or algorithm, sample size, candidate predictors, outcome measurement, missing data, performance metrics, model evaluation methods, clinical utility, model presentation, and interpretation. The full extracted data is available as a supplementary file (Appendix S1).

### Risk of bias (quality) assessment

The Prediction model Risk Of Bias ASsessment Tool (PROBAST + AI) [38] was used for assessing quality (risk of bias) and applicability of included studies. This recently published tool has not been previously applied to prognostic models in this field. Here, we applied it to provide integrated evaluation of regression- and AI-based prediction models across development and evaluation phases. PROBAST + AI has two parts: model development and model evaluation with both parts containing four domains (participants and data sources, predictors, outcome, and analysis). There are 34 signalling questions, 16 for model development and 18 for model evaluation. Signalling questions for model development are rated as yes (Y), probably yes (PY), probably no (PN), no (N), no information (NI), and in some cases not applicable (NA). Y or PY indicate high quality, and any rating of N or PN flags concerns regarding quality. For model evaluation, a similar rating process is followed; Y or PY indicate low risk of bias and questions rated as N or PN indicate a high risk of bias in that domain. The tool also has six applicability items, three each for model development and model evaluation.

The quality of included studies in this review was assessed independently by two reviewers (NBY and YB), and disagreements resolved through discussion. Assessors first completed PICOTS information of included studies, and extracted the relevant information indicated in TRIPOD + AI [39]. Secondly, they classified the type of prediction model assessment as ‘development only’, ‘evaluation only’, or ‘combination’. The third step used signalling questions in each domain of the PROBAST + AI to judge concerns regarding quality (for model development) and risk of bias (for model evaluation) as ‘low’, ‘high’, or ‘unclear’ against the review question. Similarly, the first three domains were rated for concerns of applicability (low/high/unclear). In the final step, overall judgment was made regarding quality and applicability of model development and risk of bias, and applicability of model evaluation processes. The completed PROBAST + AI is supplemented here (Appendix S2).

### Data synthesis and presentation

A narrative synthesis approach summarised the review results, and tables and figures created to present the results. Modelling methods, sample size, predictor selection strategies, missing data handling, and validation approaches were summarised. Performance metrics including model calibration and discrimination were summarised. Meta-analysis of model performance was not appropriate due to substantial heterogeneity in follow-up durations (2–30 years), predictor mix (5 to > 1100), and lack of externally validated models (only one). These violate core assumptions for pooling evaluation metrics across prediction models. Consistent with expert-guidance [10, 29], quantitative aggregation was deemed inappropriate in our case. Risk of bias and applicability were summarised for model development, model evaluation, and overall prediction model using traffic light plots.

## Results

### Study selection

**Fig. 1:**
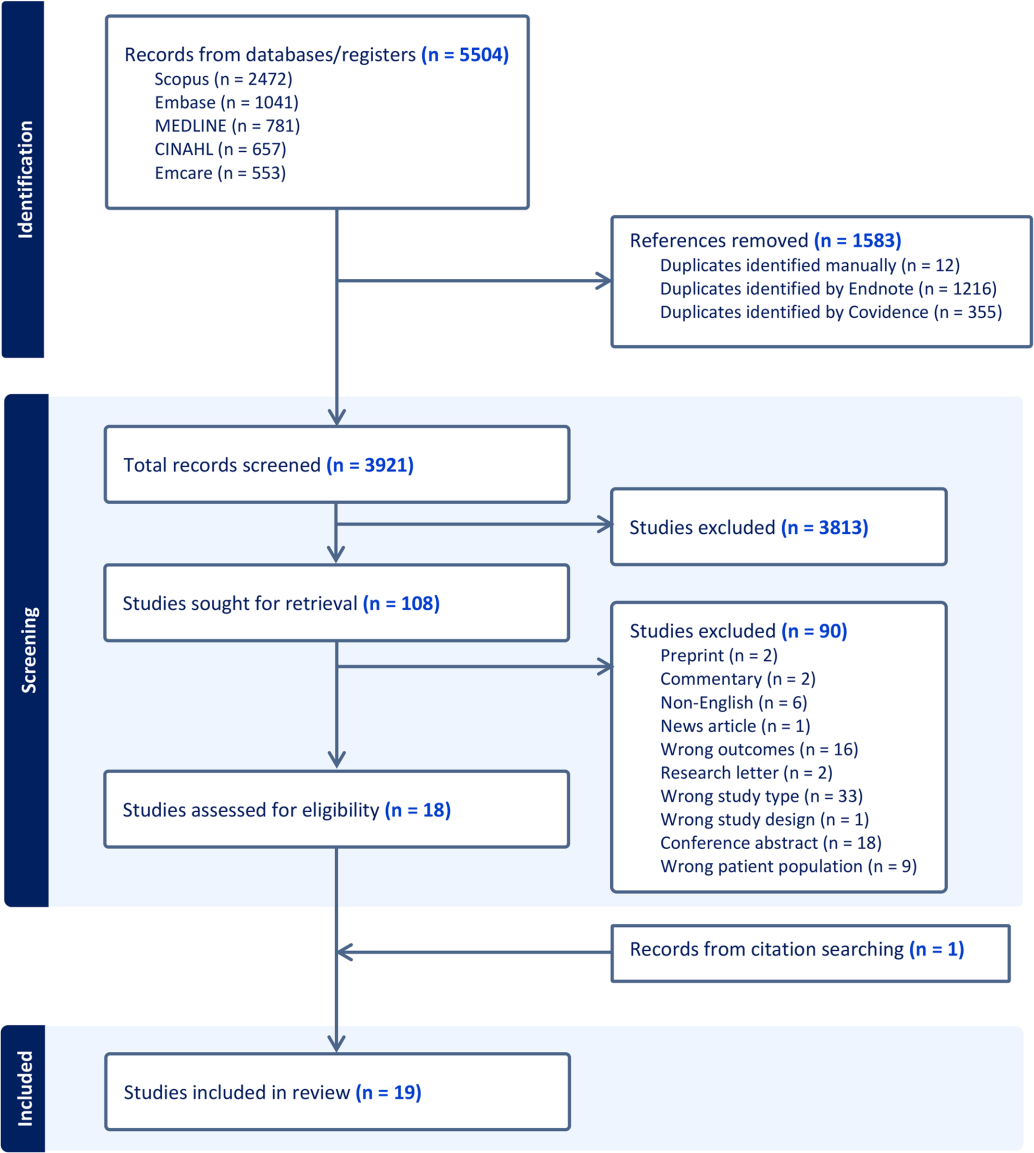
Flow diagram describing systematic literature search of prognostic studies predicting type 2 diabetes in women with history of gestational diabetes

A total of 5504 records were retrieved from five databases, and 1583 duplicates were removed through software’s automation system and manual identification. Among the 3921 articles eligible for title and abstract screening, 3813 were irrelevant. In the full text screening stage, 108 were eligible, 90 were excluded and 19 studies [20–23, 40–54] were included for the review (Fig. 1).

### Characteristics of included studies

The review identified 19 studies with 20 models. In the studies included, more than half (10/19) of these studies used a prospectively collected data [23, 40, 41, 43, 44, 48–50, 52, 54] and models from 15 studies were developed at hospital settings [20, 23, 40–42, 44–52, 54]. In terms of geographic distribution, the United States of America [21, 40, 45, 47, 52] and the Asian region [20, 22, 46, 51] each contributed five and four studies, respectively. Europe [23, 42, 43, 53, 54] and Australia [44, 48–50], contributed five and four studies, respectively. One study was multinational [41]. Follow-up duration of the studies ranged from 2 years [45] to 30 years [41], with majority of the studies ≥5 years of follow-up. Average age of the participants included in the studies ranges was 26.9 [41] to 41.3 years [21] (Table 2).

**Table 2:** Characteristics of prognostic studies predicting T2D following GDM

Author, year	Source of data	Study setting	Study region	Participant characteristics	
				Age (median or mean)	Follow-up (years)
Lin, 2011 [51]	Retrospective cohort	Hospital-based	China Taiwan	NA	NA
Kwak, 2013 [46]	Retrospective cohort	Hospital-based	South Korea	31.5 (4.1)	6.3
Lappas, 2015 [50]	Prospective cohort	Hospital-based	Australia	35.11 (31.62, 37.67)	10
Lappas, 2016 [49]	Prospective cohort	Hospital-based	Australia	35.11 (31.62, 37.67)	10
Allalou, 2016 [40]	Prospective cohort	Hospital-based	USA	33.3 (5.2)	4
Ko¨hler, 2016 [23]	Prospective cohort	Hospital-based	Germany	31.4 (28.53, 34.77)	20
Ignell, 2016 [54]	Prospective cohort	Hospital-based	Sweden	35.4 (28.8, 38.2)	5
Li, 2018 [22]	Retrospective cohort	Community-based screening and hospital follow-up	China	30.0 (3.8)	5
Lappas, 2019 [48]	Prospective cohort	Hospital-based	Australia	35.35 (31.48, 37.95)	10
Khan, 2019 [45]	Nested case-control	Hospital-based	USA	34.8 (5.1)	2
Lai, 2020 [47]	Nested case-control	Hospital-based	USA	33.2 (5.2)	8
Joglekar, 2021 [44]	Prospective cohort	Hospital-based	Australia	36.0 (33.1, 41.7)	10
Man, 2021 [21]	Randomised trial	Clinic setting	USA	41.32 (8.9)	3
Ilari, 2022 [43]	Prospective cohort	Clinic setting	Austria	36.6 (4.7)	7
Wang, 2023 [52]	Prospective cohort	Hospital-based	USA	30.22 (6.85)	20
Belsti, 2024 [20]	Randomised trial	Hospital-based	India, Sri Lanka, Bangladesh	30.1 (5.0)	3
Choi, 2024 [41]	Prospective cohort	Hospital-based and population-based cohorts	UK, Korea, Mexico, Multi-national	31.1 (5.3) *	30
Chung, 2024 [42]	Retrospective cohort	Hospital-based	Sweden	31.3 (28.4, 36.7)	10
Zöllner, 2025 [53]	Retrospective cohort	Community- and primary care-based	UK	31.73 (5.24)	10

*NA* Not Available, *USA* United States of America, *UK* United Kingdom; * pooled mean and standard deviation

### Modelling approach and sample size characteristics

Logistic regression (*n* = 9) was the most frequently employed modelling approach [20, 44, 46, 48–50, 53, 54], and another five studies used Cox regression [21–23, 41, 52]. Six studies applied ML techniques [40, 42, 43, 45, 47, 51]. Random forest [42, 47] and decision tree [40, 43, 45] classifiers were frequently used among the ML algorithms. Only one study [20], showed appropriate sample size determination. Sample sizes varied widely, ranging from 75 [43] to 3203 [53] with number of events ranging from 10 to 608. The number of candidate predictors ranged from 5 to over 1100, with events per predictor spanning from 1.4 to 121.6 (Table 3).

**Table 3:** Modelling and sample characteristics of studies predicting T2D following GDM

Author, year	Modelling method	Sample size	Events *n*	Number of predictors		EPV or EPP
				Candidate	Final	
Lin, 2011 [51]	ML: AIRS	152	10	12 clinical	12	0.8
Kwak, 2013 [46]	Logistic regression	395	116	6 clinical + GRS	7	16.6
Lappas, 2015 [50]	Logistic regression	148	21	> 300 lipids + 4 clinical	7	3
Lappas, 2016 [49]	Logistic regression	148	21	6 (4 clinical + 2 biomarkers)	6	3.5
Allalou, 2016 [40]	ML: J48 DT	244	122	182 metabolites	6	20.3
Ko¨hler, 2016 [23]	Cox regression	304	110	6	4	27.5
Ignell, 2016 [54]	Logistic regression	200	67	10	3	22.3
Li, 2018 [22]	Cox regression	1263	83	5	5	16.6
Lappas, 2019 [48]	Logistic regression	148	20	9 (4 clinical + 5 proteins)	9	2.2
Khan, 2019 [45]	ML: DT	140	55	~ 1100 metabolites	7	7.9
Lai, 2020 [47]	ML: RF	658	173	188 metabolites + 2 clinical	20	8.6
Joglekar, 2021 [44]	Logistic regression	148	21	6 clinical + 754 miRNAs	7	3
Man, 2021 [21]	Cox regression	317	82	11	5	16.4
Ilari, 2022 [43]	ML: NB, DT, LR	75	17	34 clinical & model-driven	6	2.8
Wang, 2023 [52]	Cox regression	159	55	5 clinical & 209 lipids	26	2.1
Belsti (a), 2024 [20]	Logistic regression	1299	124	19 clinical	7	17.7
Belsti (b), 2024 [20]	Logistic regression	1299	124	19 clinical	4	31
Choi, 2024 [41]	Cox regression	1895	363	6 clinical + PRS	6	60.5
Chung, 2024 [42]	ML: RF, LR, GB	213	70	49 clinical + 428 proteins	49 clinical + proteins	1.4
Zöllner, 2025 [53]	Logistic regression	3203	608	5 (4 clinical + PRS)	5	121.6

*AIRS* artificial immune recognition system, *DT* decision tree, *EPP* event per parameter, *EPV* event per variable, *GB* gradient boosting, *GRS* Genetic Risk Score, *J48 DT* J48 Decision Tree algorithm, *LR* logistic regression, *ML* machine learning, *miRNAs* Micro ribonucleic acid, *NB* naïve bayes, *PRS* Polygenic Risk Score, *RF* random forest

### Predictor selection and missing data handling methods

Five studies selected candidate predictors based on prior knowledge [20, 21, 23, 41, 46], while six included all available predictors [40, 43, 44, 47, 51, 53], and the remaining studies selected predictors based on univariable associations [22, 45, 48–50, 54]. Final predictor selection methods included least absolute shrinkage and selection operator (LASSO) [20, 23, 44, 52], backward elimination [22, 48–50, 54], stepwise selection [21, 46], and ML-based feature selection [40, 42, 43, 45, 51]. While several models did not report the number of missing data or methods used to handle them, the approaches identified included complete case analysis [23, 41, 44, 48–50], single imputation [42, 47], and multiple imputation [20, 53] (Table 4).

**Table 4:** Predictor selection and missing data handling methods of studies predicting T2D following GDM

Author, year	Selection of candidate predictors	Selection of final predictors	Missing data	
			*n* (%)	Handling method
Lin, 2011 [51]	All available predictors	AIRS immune mechanisms	NI	NI
Kwak, 2013 [46]	Prior knowledge	Stepwise selection	NI	NI
Lappas, 2015 [50]	Univariable associations	Backward elimination	44	Complete-case analysis
Lappas, 2016 [49]	Univariable associations	Backward elimination	50	Complete-case analysis
Allalou, 2016 [40]	All available predictors	Attribute selection and pruning trees	NI	NI
Ko¨hler, 2016 [23]	Prior knowledge	LASSO selection	47	Complete-case analysis
Ignell, 2016 [54]	Univariable associations	Backward elimination	NI	NI
Li, 2018 [22]	Univariable associations	Backward elimination	NI	NI
Lappas, 2019 [48]	Univariable associations	Backward elimination	53	Complete-case analysis
Khan, 2019 [45]	Univariable associations	Filter classifier with cross-validation	NI	NI
Lai, 2020 [47]	All available predictors	Pre-specified model (not selection)	56	Single imputation
Joglekar, 2021 [44]	All available predictors	LASSO selection	45	Complete case-analysis
Man, 2021 [21]	Prior knowledge	Stepwise selection	NI	NI
Ilari, 2022 [43]	All available predictors	Decision tree feature selection	NI	NI
Wang, 2023 [52]	Unclear	LASSO selection	NI	NI
Belsti (a), 2024 [20]	Prior knowledge	LASSO selection	NI	Multiple imputation
Belsti (b), 2024 [20]	Prior knowledge	LASSO selection	NI	Multiple imputation
Choi, 2024 [41]	Prior knowledge	Pre-specified model	NI	Complete case-analysis
Chung, 2024 [42]	Feature importance/permutation	Random forest feature ranking	< 5% per feature	Single imputation
Zöllner, 2025 [53]	All available predictors	Prespecified and Performance-based PRS selection	BMI (9.2%), Parity (3.6%)	Multiple imputation

*AIRS* artificial immune recognition system, *BMI* body mass index, *LASSO* least absolute shrinkage and selection operator, *PRS* polygenic risk score, *NI* no information

### Model performance measures

Internal validation was performed in most models (*n* = 13) using cross-validation [20, 21, 42, 43, 45, 50–52], bootstrapping [20, 23, 44], or random data splitting [40, 47]; two models employed both cross-validation and bootstrap approaches [20]. Only one study validated the model externally [42]. All but one study reported discrimination using c-statistic, with or without the area under the curve (AUC) graph. However, five models [21, 23, 42, 43, 50] did not report 95% confidence interval estimates for the c-statistics. Most models reported moderate to high discrimination performances, AUC value ranging from 0.67 [42, 53] to 0.92 [44, 45]. Calibration measure using slope or plots was reported in only four models [20, 22, 23]. Few studies also reported mean absolute error [20], Hosmer-Lemeshow test [46], and likelihood ratio test [21] as calibration measures. Overall model performance was less frequently evaluated, with only two models reported Brier score or R-squared values [20, 23]. Five models presented model equation or a scoring system [20, 21, 23, 54] and one model constructed a nomogram for clinical use [22] (Table 5).

**Table 5:** Model evaluation characteristics of studies predicting T2D following GDM

Author, year	Type of validation	Model performance			Model presentation/alternative
		Calibration	Discrimination AUC (95% CI)	Overall measure	
Lin, 2011 [51]	IV: Cross-validated	None	NR	NR	None
Kwak, 2013 [46]	None	None	0.77 (0.72, 0.83)	NR	None
Lappas, 2015 [50]	IV: Cross-validated	None	0.86*	NR	None
Lappas, 2016 [49]	None	None	0.89 (0.81, 0.97)	NR	None
Allalou, 2016 [40]	IV: Random split data	None	0.77 (0.67, 0.87)	NR	None
Ko¨hler, 2016 [23]	IV: Bootstrap	Slope: 1.13	0.75*	R^2^: 0.23, 0.26, 0.33**	Scoring system
Ignell, 2016 [54]	None	None	0.91 (0.86, 0.95)	NR	Scoring system
Li, 2018 [22]	None	Calibration plot	0.83 (0.78, 0.87)	NR	Nomogram
Lappas, 2019 [48]	None	None	0.82 (0.70, 0.94)	NR	None
Khan, 2019 [45]	IV: Cross-validated	None	0.92 (0.89, 0.95)	NR	None
Lai, 2020 [47]	IV: Random split data	None	0.88 (0.82, 0.94)	NR	None
Joglekar, 2021 [44]	IV: Bootstrap	None	0.92 (0.84, 1.00)	NR	None
Man, 2021 [21]	IV: Cross-validated	None	0.69*	NR	Scoring system
Ilari, 2022 [43]	IV: Cross-validated	None	LR: 0.88, NB: 0.83, DT: 0.79	NR	None
Wang, 2023 [52]	IV: Cross-validated	None	0.71 (0.63, 0.80)	NR	None
Belsti (a), 2024 [20]	IV: Cross-validated + Bootstrap	Calibration plot; Slope: 0.94	0.76 (0.72, 0.80)	Brier score: 0.078	Scoring system
Belsti (b), 2024 [20]	IV: Cross-validated + Bootstrap	Calibration plot; Slope: 0.98	0.85 (0.81, 0.88)	Brier score: 0.07	Scoring system
Choi, 2024 [41]	None	None	0.80 (0.76, 0.84)	NR	None
Chung, 2024 [42]	IV: Cross-validated; EV: Geographical	None	0.67*	NR	None
Zöllner, 2025 [53]	None	None	0.67 (0.65, 0.69)	NR	None

*AUC* area under the curve, *IV* internal validation, *EV* external validation, *NB* naïve bayes, *DT* decision tree, *LR* logistic regression; * confidence intervals were not reported; ** 5, 10, and 15 years postpartum respectively; *NR* not reported

### Predictors utilised in the models

**Fig. 2:**
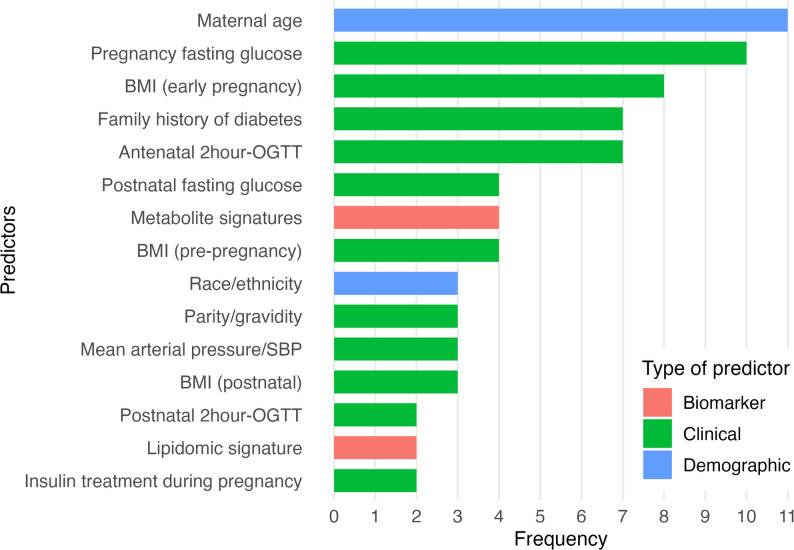
Top 15 predictors used in the included prognostic models predicting T2D following GDM (abbreviations: BMI body mass index, SBP systolic blood pressure; OGTT oral glucose tolerance test)

Clinical and demographic variables were most frequently employed. Maternal age was the most commonly used predictor (*n* = 11), followed by pregnancy fasting glucose (*n* = 10) and early pregnancy BMI (*n* = 8). Metabolic and lipidomic signatures (i.e., specific lipid profiles associated with metabolic states or disease risk) were also utilised by some studies. Genetic and other biomarker related predictors were rarely used (Fig. 2).

### Quality assessment

#### Quality and applicability assessment in model development

**Fig. 3:**
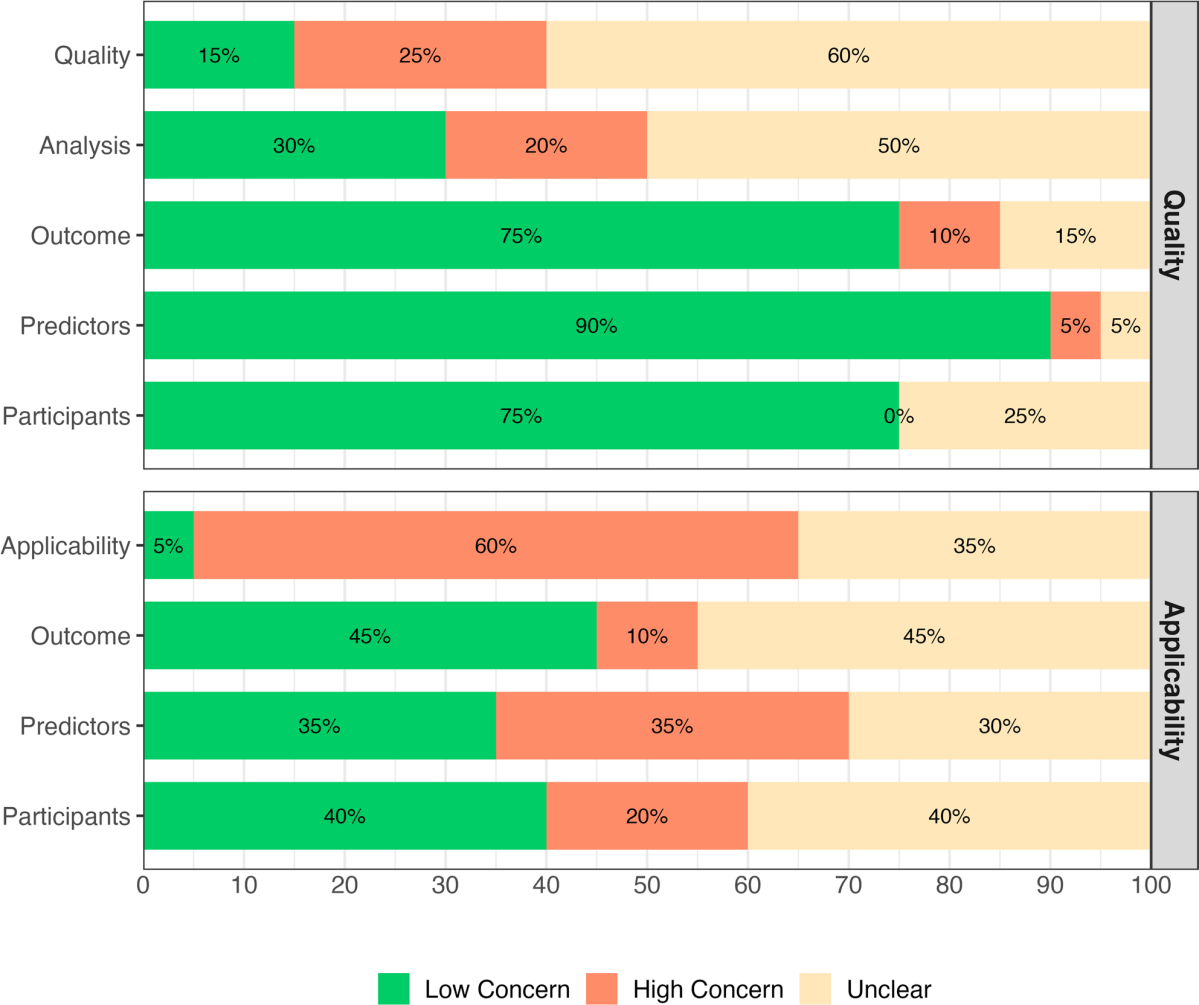
Summary of quality and applicability assessment in model development for studies predicting T2D following GDM

In model development, the quality concern of the included studies showed considerable variability. While concerns were generally low for predictors (90%), participants (75%), and outcomes (75%), analysis and overall quality domains had higher proportions of ‘unclear concerns’ (50% and 60% respectively). Similarly, high concern was most notable in analysis (20%) and overall quality (25%) domains. Applicability of model development showed substantial concerns. The overall applicability was rated as ‘high concern’ in 60% of the studies, with predictors (35%) and participants (20%) contributing the most for high concern. Unclear concern was common across all applicability domains, particularly for outcome (45%) and participants (40%). Similarly, 45% of outcome and 40% of participants domains were rated low concern for applicability (Fig. 3). The findings show limitations in statistical analysis of models and applicability assessment weaken confidence in the developed models.

#### Risk of bias and applicability assessment in model evaluation

**Fig. 4:**
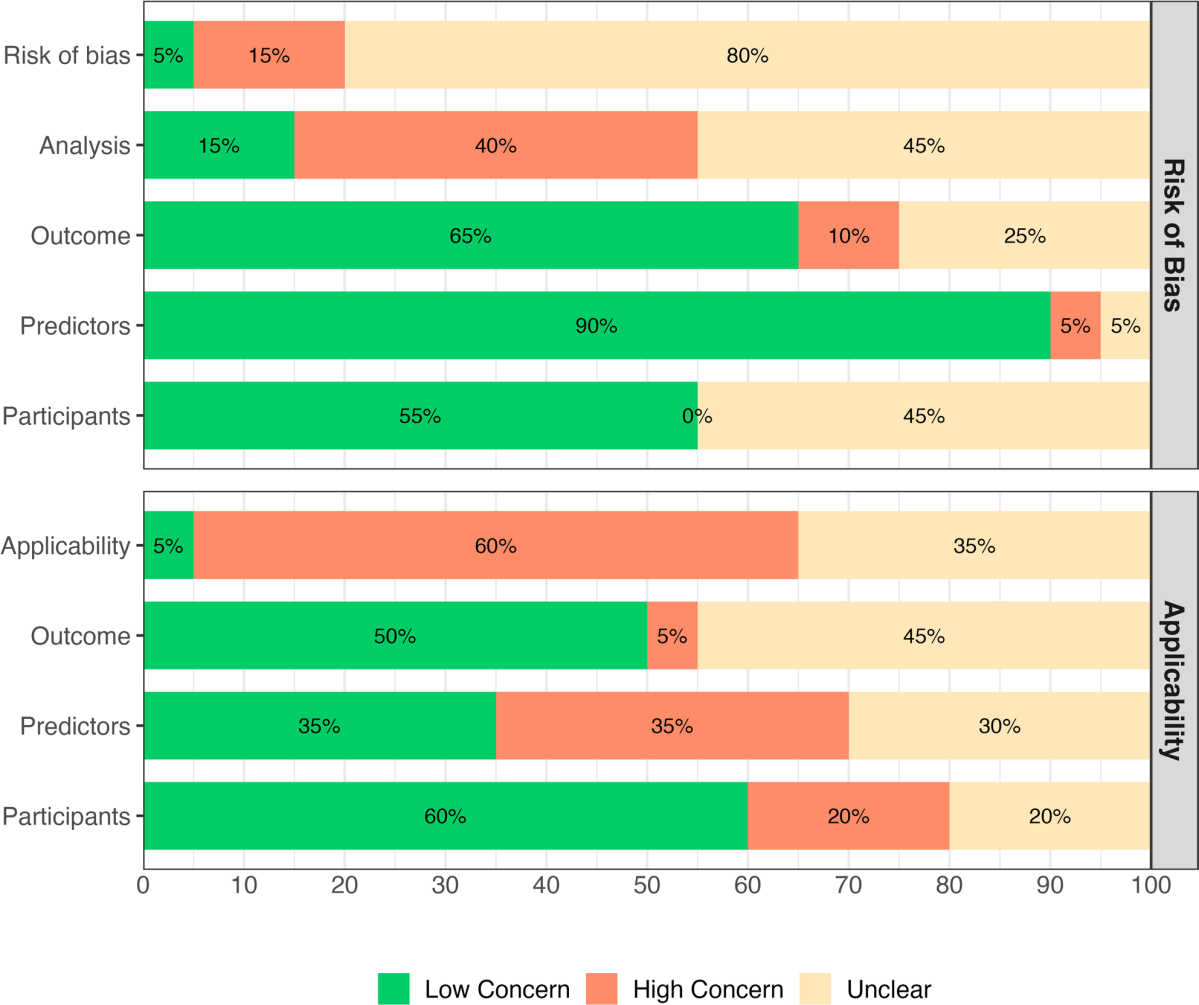
Summary of risk of bias and applicability assessment in model evaluation for studies predicting T2D following GDM

Model evaluation assessment showed moderate to considerable concerns regarding risk of bias and applicability. Overall risk of bias was judged as unclear and high concerns in 80% and 15% of the studies, respectively. Majority of the studies in participants (75%), predictors (90%), and outcome (75%) domains were rated as low concern for risk of bias. Additionally, 40% of the studies flagged high concern in analysis domain. 60% of the studies were rated as high concern for overall applicability. Predictors and outcome domains of applicability were rated as low concern in 40% and 45% of the studies, respectively. Similar percentage of studies had unclear concern for applicability in these domains (Fig. 4). Despite of low risk of bias in several domains, limitations in analysis method and concern in applicability reduced robustness of the models.

#### Overall quality assessment of the included prediction models

**Fig. 5:**
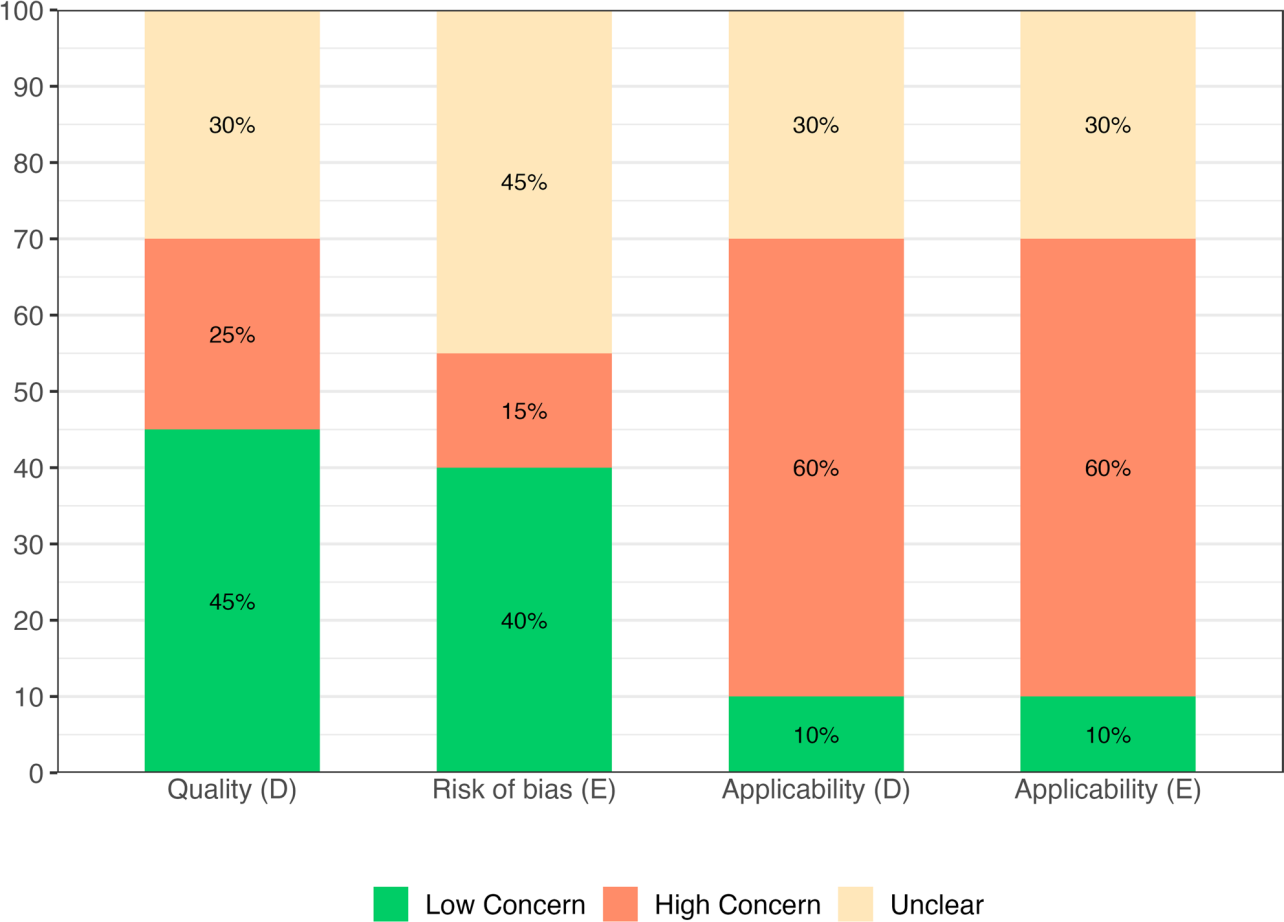
Summary of overall quality assessment of the included prediction models for T2D following GDM (D: development, E: evaluation)

While quality and risk of bias assessment showed low to fair concerns in both development and evaluation phases, substantial concerns were noted for applicability in both phases of the prediction models. Quality in model development was rated as low concern in 45% of studies, though 25% were judged to have high concern and 30% remained unclear. Variable concerns were noted for risk of bias assessment for model evaluation, with 40% low, 15% high and 45% unclear concerns. In terms of applicability, 60% of studies were judged as high concern and only 10% as low concern in each phase (Fig. 5). The use of PROBAST + AI enabled us identifying applicability concerns that were not previously appreciated, amplifying the practical limitations of current prediction tools for real-world application.

#### Optimistic performance and limited applicability of the models

Many models demonstrated acceptable discriminative performance (AUC 0.67–0.92), but with high applicability concerns. One possible reason for this could be due to risk of overfitting, given multiple models had small event per predictors ranging from as low as 1.4 to 121.6 (Table 3). Validation choice such as split-sample approaches can also inflate discrimination. Another point is handling of missing data, where some applied complete case analyses without justifying pattern the of missingness (Table 4). This may result bias in predictor effects and distort discrimination estimates if the missingness is informative (not random). Furthermore, underreporting of calibration and clinical utility measures limits the accuracy and applicability of predicted risk.

## Discussion

This review synthesises prognostic models for T2D following GDM By applying the updated PROBAST + AI, TRIPOD-SRMA, and CHARMS, this work presents the first comprehensive synthesis of regression- and AI-based, omics-integrated, and biomarker-augmented models through a unified appraisal structure. These enabled us to identify previously unreported limitations, including applicability, sample size justification, and clinical usefulness, that were not captured in earlier reviews. While several models reported model discrimination measure and demonstrated low concern in participants, predictors, and outcome domains, the PROBAST + AI evaluation revealed substantial concerns in analysis domain and high applicability concerns. Hence, methodological quality of the models can be considered acceptable only in selected domains.

Already mentioned in the introduction is that two recent systematic reviews did not consistently adhere to established methodological standards. In the context of our findings, this reinforces the need for more rigorous and transparent evidence synthesis to ensure that conclusions about ML‑based prediction of T2D after GDM are both methodologically sound and clinically meaningful. By addressing these limitations, our review provides a more reliable foundation for understanding current model performance and highlights key priorities for strengthening future prediction research in this field. Importantly, beyond our adherence to best‑practice methodology, our review identified six additional eligible studies that were not captured previously, while the earlier reviews had incorrectly included two ineligible studies. These distinctions substantially improve the accuracy and relevance of the available evidence. Consequently, our work offers a clearer and more valid representation of the current state of ML‑based risk prediction after GDM, serving as a practical example of how future researchers should conduct systematic evaluations of clinical prediction models and supporting the development of more robust, clinically applicable tools.

Most models were developed in high-income settings, with only one study used data from three Asian countries [20]. Given the lack of developed or externally validated models in developing countries, existing prognostic models for T2D following GDM could not be generalised and applicable to these populations, underscoring the need for context-specific model development and validation.

Maternal age, BMI (measured at early pregnancy), family history of diabetes, and Oral Glucose Tolerance Test (OGTT) were the most frequently used predictors. Some models integrating metabolic and lipidomic markers reported improved discrimination, demonstrating potential omics-enhanced prediction [55, 56]; however, given the methodological limitations, this potential remains preliminary. Predictor selection commonly relied on using all available predictors, prior knowledge, or univariable associations, with final selection refined with LASSO, backward elimination, or ML-based feature reduction. Selection techniques carry risks; univariable screening may omit clinically relevant factors [13, 24] and ML-based variable selection methods often lack interpretability [14]. Penalisation methods have been suggested to reduce risk of overfitting, particularly in datasets with few events [12, 57]. These findings highlight the need to balance feasibility, statistical performance, and clinical interpretability when selecting predictors.

Across the models, logistic regression was most frequently used, likely due to its interpretability and familiarity in binary classification [14]. Evidence suggests logistic regression performs comparably to ML in predicting conditions with low incidence and simple clinical predictors [58]. The ML models are developed using modest samples, diverse predictor mix, and limited model calibration and external validation, conditions that pose optimistic performance than generalisability. Advances have also been made to address the “black box” nature of ML [59, 60], however, none of the included studies used these.

Comprehensive reporting of methodology is a critical component of clinical prediction models [10, 39], yet sample size calculation is an often-overlooked aspect [61]. With the exception of one study [20], none of the included studies reported sample size calculation despite wide variability in events per predictor. In agreement with this finding, a systematic review revealed many prediction models are developed without sample size calculations [62]. Inadequate sample size leads to overfitting, unstable estimates, and poor clinical utility [61, 62]. Sample size determination methods are available [63–67]. Moving forward, methodological transparency and reproducibility can be improved through evaluating and adapting these existing sample size guides and the TRIPOD + AI. Similarly, reporting on missing data handling was insufficient, with complete-case analysis reported in some without justifying missingness pattern, potentially biasing validity of results [12] and inflating model discrimination. Rigorous and transparent handling of missing data, including multiple imputation [68] is essential to ensure trustworthiness and generalizability.

While all models reported discrimination, some failed to present confidence intervals. Calibration and overall performance measures were largely unreported, and clinical utility was rarely assessed. Internal validation was common and likely improved robustness yet, external validation was conducted in only one study, compromising generalisability. These represent critical gaps that future work should address through tailored frameworks aiming at identification and mitigation of ‘spin’ practices [69] and external validation efforts [70]. As indicated in best practice guides (CHARMS, TRIPOD + AI), transparent reporting of these measures can offer clinicians a more intuitive understanding of model outputs and facilitate integration into clinical decision-making.

Consistent with a previous systematic review, analysis domain was the most frequently rated as high risk of bias due to factors such as inadequate event per predictors, poor handling of missing data, and improper evaluation of overfitting [71]. High concern ratings for applicability due to similar factors suggest these models may not be readily transferable to more diverse populations. These highlight the need for more rigorous and transparent reporting aligned with the existing best-practice guides mentioned earlier [24, 38, 39]. It is also worth noting the importance of interpreting PROBAST + AI results in a domain-specific way. In this work, many models scored well in methodological quality but poorly in applicability, partly due to limited external validation, small sample size, specialised predictors not routinely available, and underreporting of other key metrics. Hence, the findings are not contradictory, but reflect dimensions assessed in PROBAST + AI tool.

### Implications for clinical practice, policy and research

Appropriately developed and evaluated risk prediction tools can address clinical practice challenges among women with prior GDM, including low risk perception and limited adherence to postpartum screening, by providing individualised risk estimates and improving communication with providers. Integration of prediction tools into routine care could help identify high-risk women who would benefit from lifestyle interventions such as healthy diet and physical exercise, which can prevent or delay onset of T2D [72]. Embedding these tools in patient management protocols and digital platforms may support implementation and shared decision-making, ultimately enhancing personalised care in this high-risk group. Importantly, substantial methodological improvements are required to the current models before clinical deployment can be recommended.

### Strengths and limitations

A strength is that we included all available prognostic models on T2D following GDM, regardless of the setting and modelling methods. We summarise characteristics of developed or evaluated models, with particular focus on calibration, overall measure, and clinical utility measures - areas not addressed in earlier reviews [30, 33]. Additionally, this review is the first to apply up-to-date best-practice guides such as the PROBAST + AI for the prediction field to assess regression- and machine learning-based prognostic models. We did not undertake meta-analysis because experts agree that this is inappropriate when less than five external validation studies not available and assumptions violate for pooling [10, 29]. Meta-analysis when done on inappropriately selected or non-comparable samples enables inappropriate comparisons to be made, thereby not assisting the discipline to progress using the correct available evidence.

Optimistic but non-generalisable performance of the models due to high concern for applicability might arise from overfitting risk due to imbalance of events per predictor, validation choice, missing data handling, and underreporting of key calibration and clinical utility measures. Specifying the exact gestational week would strengthen clarity, however, the original studies included in our review typically reported BMI as measured in “early pregnancy” or “at the start of pregnancy” without providing a specific gestational age. As such, we were unable to extract an exact gestational week. In general, early‑pregnancy BMI is commonly assessed during the first trimester, typically around or before 12 weeks’ gestation, consistent with how this is defined in the literature [73]. Although planned, we were unable to conduct a meta-analysis of performance metrics due to the lack of external validations and considerable heterogeneity between the studies. No examples of models integrating AI and ML with causal inference approaches were found, however, these new approaches have been proposed to enhance interpretability and warrant future research.

## Conclusion

This review highlights methodological practices of models predicting T2D following GDM, employing statistical and AI-based approaches. Clinical and demographic predictors such as age, BMI and fasting glucose were commonly used, with some studies integrating biomarkers and omics data. Key methodological concerns include inadequate handling of missing data, underreporting of calibration and overall performance metrics, and lack of clinical utility evaluation. Although internal validation was common, lack of sufficient externally validated studies and high applicability concerns limits generalisability and readiness for clinical adoption. This review provides the first integrated synthesis of regression- and AI-based models using the recently updated PROBAST + AI tool, highlighting insights not captured in earlier works. Future studies should adhere to established guidelines like TRIPOD + AI, PROBAST + AI, and CHARMS, to ensure transparent reporting, and validate models across diverse populations.

## Supplementary Information

Supplementary Material 1.

Supplementary Material 2.

Supplementary Material 3.

Supplementary Material 4.

Supplementary Material 5.

## Data Availability

All data generated or analysed during this study are included as supplementary file.

## Abbreviations

AUC: Area under the receiver operating characteristic curve
CHARMS: CHecklist for critical Appraisal and data extraction for systematic reviews of prediction Modelling Studies
TRIPOD + AI: Transparent Reporting of a multivariable prediction model of Individual Prognosis Or Diagnosis Plus Artificial Intelligence
GDM: Gestational diabetes mellitus
LASSO: Least absolute shrinkage and selection operator
ML: Machine learning
OGTT: Oral glucose tolerance test
PROBAST + AI: Prediction model Risk Of Bias ASsessment Tool Plus Artificial Intelligence
TRIPOD-SRMA: Transparent Reporting of a multivariable prediction model of Individual Prognosis Or Diagnosis checklist for Systematic Reviews and Meta-Analysis
T2D: Type 2 diabetes
