# Supplementary material for: Traditional statistics and artificial intelligence-based prognostic models for predicting type 2 diabetes mellitus after gestational diabetes: a systematic review
Source: Diagn Progn Res. 2026 Apr 20;10:12. doi: 10.1186/s41512-026-00229-8 (PMC13094168; doi:10.1186/s41512-026-00229-8)
Supplement: Supplementary file 2 — Supplementary Material 2. [file 41512_2026_229_MOESM2_ESM.docx]

**Table S2** Search strategy for Traditional statistics and artificial intelligence-based prognostic models for predicting type 2 diabetes mellitus after gestational diabetes: a systematic review

| **Database** | **Date of search** | **Search terms** | **Results** |
| --- | --- | --- | --- |
| Ovid MEDLINE(R) | 12/05/2025 | (exp Diabetes, Gestational/ or diabetes in pregnancy.mp. or gestational diabetes.mp. or GDM.mp.) adj5 (previous* or follow* or transition or progression or outcome).mp. AND*predict.mp. OR model*.mp. OR biomarker*.mp. OR exp risk/ OR clinical prediction.mp. OR Clinical Decision Rules/ OR prediction rule.mp. OR prognosis/ OR nomograms/ OR prognostic model.mp. OR prognostic score.mp. OR prognostic index.mp. OR indices.mp. OR risk score.mp. OR risk calculator.mp. OR reclassification.mp. OR discrimination.mp. OR stratification.mp. OR Calibration/ OR exp "predictive value of tests"/ OR roc curve/ OR area under curve/ OR models, statistical/ OR (c statistic OR c-statistic).mp. OR Validation Study/ OR multivariable.mp. OR (artificial intelligence OR AI).mp. OR exp Machine Learning/ OR exp Algorithms/ AND (type 2 diabetes OR T2DM).mp. OR exp Diabetes Mellitus, Type 2/ OR postpartum diabetes.mp. OR type two diabetes.mp. OR Glucose Intolerance/ OR postpartum glucose intolerance.mp. | 753 |
| Ovid EMBASE | 12/05/2025 | (exp Diabetes, Gestational/ or diabetes in pregnancy.mp. or gestational diabetes.mp. or GDM.mp.) adj5 (previous* or follow* or transition or progression or outcome).mp. AND *exp prediction/ OR predict.mp. OR model*.mp. OR biomarker*.mp. OR risk/ OR clinical prediction.mp. OR exp clinical decision rule/ OR exp prognosis/ OR nomograms/ OR prognostic model.mp. OR prognostic score.mp. OR prognostic index.mp. OR indices.mp. OR risk score.mp. OR risk calculator.mp. OR reclassification.mp. OR discrimination.mp. OR stratification.mp. OR Calibration/ OR exp predictive value/ OR exp receiver operating characteristic/ OR exp area under the curve/ OR exp statistical model/ OR exp Youden index/ OR (c statistic OR c-statistic).mp. OR Validation Study/ OR exp multivariate analysis/ OR multivariable.mp. OR (artificial intelligence OR AI).mp. OR exp machine learning/ OR exp Algorithm/ AND exp non insulin dependent diabetes mellitus/ OR postpartum diabetes.mp. OR type two diabetes.mp. OR glucose intolerance/ OR postpartum glucose intolerance.mp. | 928 |
| Ovid Emcare | 12/05/2025 | (exp gestational diabetes/ or GDM.mp.) adj5 (previous* or follow* or transition or progression or outcome).mp. AND *exp prediction/ OR predict.mp. OR model*.mp. OR biomarker*.mp. OR exp risk/ OR clinical prediction.mp. OR exp clinical decision rule/ OR prognosis/ OR nomogram/ OR prognostic model.mp. OR prognostic score.mp. OR prognostic index.mp. OR indices.mp. OR risk score.mp. OR risk calculator.mp. OR reclassification.mp. OR discrimination.mp. OR stratification.mp. OR Calibration/ OR exp predictive value/ OR exp receiver operating characteristic/ OR exp area under the curve/ OR exp statistical model/ OR (c statistic OR c-statistic).mp. OR Validation Study/ OR multivariable.mp. OR (artificial intelligence OR AI).mp. OR exp machine learning/ OR exp algorithm/ AND exp non insulin dependent diabetes mellitus/ OR postpartum diabetes.mp. OR type two diabetes.mp. OR glucose intolerance/ OR postpartum glucose intolerance.mp. | 446 |
| CINAHL | 12/05/2025 | *(MH "Clinical Prediction Rules") OR (MH "Predictive Value of Tests") OR (MH "Predictive Research") OR (MH "Prediction Models") OR (MH "Prediction Algorithms") OR (MH "Clinical Prediction Rules") OR "prediction rule" OR (MH "Predictive Validity") OR predict OR nomogram* OR reclassification OR "net reclassification improvement" OR (MH "Discrimination+") OR "decision curve" OR (MH "Prognosis+") OR "prognostic index" OR "prognostic factors" OR prognostic OR indices OR "risk scor*" OR (MH "Risk Factors+") OR "receiver operating characteristic" OR (MH "ROC Curve") OR AUC OR "area under the curve" OR (MH "Calibration") OR validation OR (MH "Validation Studies") OR AI OR (MH "Artificial Intelligence+") OR (MH "Machine Learning Algorithms") OR (MH "Machine Learning+") OR multivariable OR (MH "Multivariate Analysis+") AND (MH "Diabetes Mellitus, Type 2") OR "type 2 diabetes mellitus" OR "type two diabetes" OR "T2DM" AND (MH "Diabetes Mellitus, Gestational") OR "gestational diabetes mellitus" OR "GDM" OR "diabetes in pregnancy" | 641 |
| Scopus with Scopus AI | 12/05/2025 | ( ( TITLE-ABS-KEY ( "gestational diabetes" ) OR TITLE-ABS-KEY ( "diabetes in pregnancy" ) OR TITLE-ABS-KEY ( "GDM" ) ) ) AND ( ( TITLE-ABS-KEY ( "prediction" ) OR TITLE-ABS-KEY ( "model" ) OR TITLE-ABS-KEY ( "biomarker" ) OR TITLE-ABS-KEY ( "Clinical Decision Rules or prediction rule" ) OR TITLE-ABS-KEY ( "prognosis" ) OR TITLE-ABS-KEY ( "nomogram" ) OR TITLE-ABS-KEY ( "prognostic model" ) OR TITLE-ABS-KEY ( "prognostic index" ) OR TITLE-ABS-KEY ( "prognostic score" ) OR TITLE-ABS-KEY ( "indices" ) OR TITLE-ABS-KEY ( "risk score" ) OR TITLE-ABS-KEY ( "reclassification" ) OR TITLE-ABS-KEY ( "discrimination" ) OR TITLE-ABS-KEY ( "stratification" ) OR TITLE-ABS-KEY ( "calibration" ) OR TITLE-ABS-KEY ( "predictive value" ) OR TITLE-ABS-KEY ( "receiver operating characteristic" ) OR TITLE-ABS-KEY ( "ROC curve" ) OR TITLE-ABS-KEY ( "area under the curve" ) OR TITLE-ABS-KEY ( "AUC" ) OR TITLE-ABS-KEY ( "c statistic" ) OR TITLE-ABS-KEY ( "validation" ) OR TITLE-ABS-KEY ( "multivariable" ) OR TITLE-ABS-KEY ( "statistical model" ) OR TITLE-ABS-KEY ( "artificial intelligence or AI" ) OR TITLE-ABS-KEY ( "machine learning" ) OR TITLE-ABS-KEY ( "algorithms" ) ) ) AND ( ( TITLE-ABS-KEY ( "type 2 diabetes" ) OR TITLE-ABS-KEY ( "T2DM" ) OR TITLE-ABS-KEY ( "type 2 diabetes mellitus" ) OR TITLE-ABS-KEY ( "postpartum diabetes" ) OR TITLE-ABS-KEY ( "type two diabetes or type II diabetes" ) ) ) AND ORIG-LOAD-DATE AFT 20250512 | 2234 |
